# The association between glaucoma and risk of depression: a nationwide population-based cohort study

**DOI:** 10.1186/s12886-018-0811-5

**Published:** 2018-06-22

**Authors:** Yu-Yen Chen, Yun-Ju Lai, Jen-Pang Wang, Ying-Cheng Shen, Chun-Yuan Wang, Hsin-Hua Chen, Hsiao-Yun Hu, Pesus Chou

**Affiliations:** 1 School of Medicine, National Yang-Ming University, Taipei, Taiwan; 2 Department of Ophthalmology, Taichung Veterans General Hospital, Taichung, Taiwan; 3 Community Medicine Research Center and Institute of Public Health, National Yang-Ming University, 155, sec.2, Linong St, Taipei, 11221 Taiwan; 4 Division of Endocrinology and Metabolism, Department of Internal Medicine, Puli Branch of Taichung Veterans General Hospital, Nantou, Taiwan; 5 Department of Exercise Health Science, National Taiwan University of Sport, Taichung, Taiwan; 6 Bethel Psychiatric Clinic, Taipei, Taiwan; 7 Department of Medical Research, Taichung Veterans General Hospital, Taichung, Taiwan; 8 Institute of Biomedical Science and Rong Hsing Research Center for Translational Medicine, Chung-Hsing University, Taichung, Taiwan; 9 Division of Allergy, Immunology and Rheumatology, Department of Internal Medicine, Taichung Veterans General Hospital, Taichung, Taiwan; 10 School of Medicine, Chung-Shan Medical University, Taichung, Taiwan; 11 Department of Education and Research, Taipei City Hospital, Taipei, Taiwan

**Keywords:** Glaucoma, Depression, Risk factors, National Health Insurance Research Database, Cohort study

## Abstract

**Background:**

Previous cross-sectional studies revealed a higher prevalence of depression among glaucoma patients. However, cohort studies were in lack to build the risk of incident depression after the diagnosis of glaucoma. The aim of our study was to investigate the association between glaucoma and the subsequent risk of developing depression and to assess risk factors associated with depression in glaucoma patients.

**Methods:**

A population-based retrospective cohort study using the Taiwan National Health Insurance Research Database was conducted from January 1, 2001 through December 31, 2011. Glaucoma patients (*n* = 8777) and age- and gender-matched control subjects without glaucoma (*n* = 35,108) were enrolled in the study. Kaplan-Meier curves were generated to compare the cumulative hazard of subsequent depression between the glaucoma and control groups. A Cox regression analysis estimated the crude and adjusted hazard ratios (HRs) for depression. Risk factors leading to depression were investigated among the glaucoma patients.

**Results:**

Glaucoma patients had a significantly higher cumulative hazard of depression compared to the control group (*p*-value < 0.0001). The Cox regression model indicated that the glaucoma group had a significantly higher risk of depression (adjusted HR = 1.71). Within the glaucoma group, significant risk factors for depression included age, female, low income, substance abuse, and living alone. However, the use of β-blocker eye drops and the number of glaucoma medications were not significant risk factors for depression.

**Conclusion:**

Patients with glaucoma are at significantly greater risk of developing depression. Among glaucoma patients, age, female, low income, substance abuse, and living alone were significant risk factors for depression.

## Background

Glaucoma is the most common cause of irreversible blindness [1]. Glaucoma patients must cope with visual impairment, the anxiety associated with blindness, long-term follow-up and a heavy economic burden. In addition, prior studies have shown an association between β-blocker eye drops (a common glaucoma treatment) and depression [2, 3]. In all, there are many factors associated with glaucoma that may lead to depression.

Previous studies have shown that the prevalence of depression among the glaucoma patients is higher than that among the non-glaucoma individuals [4–8]. The risk factors associated with depression among glaucoma patients have also been identified [6, 8–14]. However, the majority of the studies have been conducted in hospital settings with a small sample size. Moreover, most studies have been cross-sectional studies and as such cannot address the timeline between glaucoma and the development of depression.

To investigate the relationship of glaucoma and subsequent development of depression, we conducted a population-based, retrospective cohort study with an 11-year follow-up period. A glaucoma group and non-glaucoma group were explored through the National Health Insurance Research Database (NHIRD) in Taiwan to see whether the risk of developing depression differed between groups. In addition, risk factors associated with the development of depression among glaucoma patients were explored.

## Methods

### Data source

The National Health Insurance (NHI) program of Taiwan covers the health care services of greater than 99% of Taiwan’s 23 million residents [15]. The NHIRD is maintained by the National Health Research Institutes of Taiwan and includes inpatient as well as outpatient medical benefit claims. To ensure confidentiality, the identification of all patients in the database was encrypted prior to releasing data for research purposes [16]. Data from our study were extracted from the Longitudinal Health Insurance Database (LHID), a subset of the NHIRD that contains the original claims data of 1,000,000 beneficiaries randomly sampled from all beneficiaries of the NHI program [16]. This study was approved by the ethical committee of Yang-Ming University Hospital (2015A017). The informed consent was exempt according to the Institutional Review Board because each patient record was anonymized and de-identified prior to analysis.

### Inclusion and exclusion criteria

A retrospective cohort study of patients in the LHID with a follow-up period from January 1, 2001 to December 31, 2011 was performed. Patients with a diagnosis of glaucoma according to the International Classification of Diseases, 9th Revision, Clinical Modification Codes (ICD-9-CM code: 365.X) were selected for the glaucoma group. Date of first glaucoma diagnosis was defined as the index date. Individuals who had never been diagnosed with glaucoma were randomly selected as a control group at a ratio of 1:4, and matched with the glaucoma group on age, gender, and index year (the year of index date or enrollment). The two groups were followed until the end of 2011 to see whether they had subsequent development of depression (ICD-9-CM codes: 296.2X, 296.3X, 3004, 311). Patients with depression were diagnosed by psychiatrists under the well-acknowledged diagnostic criteria and protocol. Glaucoma subjects and controls that were diagnosed with depression or a severe mental health disorder prior to the index year were excluded from the analysis.

### Study variables

We compared the risk of developing depression among the glaucoma group and the control group. The covariates of age, gender and other risk factors for depression, including economic status and systemic diseases were adjusted. In the NHI program, insurance cost is correlated with income so that the greater the income of a patient, the more he/she pays for insurance. In addition, urbanization level of residency was also accessible. Living alone, substance abuse, and the Charlson comorbidity index (CCI), which is generally regarded as a score for severity of systemic diseases [17], were also considered covariates in our study.

### Association between topical glaucoma medication and depression

Risk factors associated with the development of depression were analyzed in the glaucoma group. The number and types of glaucoma medication, were entered into the analysis. Topical glaucoma medications were identified and classified by the National Drug Code and the Anatomic Therapeutic Chemical (ATC) code. According to the classification system, topical glaucoma medications include sympathomimetics, parasympathomimetics (pilocarpine), carbonic anhydrase inhibitors, β-blockers, prostaglandin analogs, and fixed combinations. The more degree of IOP lowering required, the greater number of glaucoma medications were prescribed. Current users were defined as those who had used medications in the past 6 months. A fixed-combination medication was counted as two medications.

### Statistical analysis

Group differences between the glaucoma group and the control group in age, gender, CCI, insurance cost, urbanization level of residency, living alone, and substance abuse were analyzed by two-sample t-tests (for continuous variables) and chi-squared tests (for categorical variables). A survival analysis using the Kaplan-Meier estimator with a log-rank test was applied to describe and compare the cumulative hazard curves of depression. The Cox proportional hazard model was used to estimate the hazard ratio (HR) for the occurrence of depression according to each variable in univariate and multivariate analyses. Variables included in the regression analysis were glaucoma, age, gender, CCI, insurance cost, urbanization level of residency, living alone, and substance abuse.

Subsequently, the cox regression was used to investigate risk factors associated with depression among glaucoma patients. Variables included for analysis were age, gender, CCI, insurance cost, urbanization level of residency, living alone, substance abuse, number of glaucoma medications, and types of glaucoma medication. All statistical operations were performed using SAS statistical package, version 9.2 (SAS Institute, Cary, NC, USA).

## Results

### Demographic characteristics of the study sample

A total of 8777 glaucoma patients and 35,108 matched controls were enrolled in the study. Table 1 displays demographics of the two groups. The mean age in both groups was 59.4 years. The glaucoma group had a higher CCI, higher insurance costs and a higher urbanization level of residency. During the 11-year follow-up period, the cumulative incidence of depression was significantly higher in the glaucoma group (5.9%) than the control group (3.2%).

**Table 1:** Characteristics of study subjects

Variable	Glaucoma group *n* = 8777	Control group *n* = 35,108	*p-*value
	*n* (%)	*n* (%)	
Age, year, (mean±SD)	59.4±13.6	59.4±13.6	1.000
Age, categorical			1.000
30–50	2228 (25.4)	8912 (25.4)	
50–60	2012 (22.9)	8048 (22.9)	
60–70	2239 (25.5)	8956 (25.5)	
≥ 70	2298 (26.2)	9192 (26.2)	
Gender			1.000
Male	4376 (49.9)	17,504 (49.9)	
Female	4401 (50.1)	17,604 (50.1)	
Charlson comorbidity index			< 0.0001
< 3	7463 (85.0)	309,339 (88.1)	
≥3	1314 (15.0)	4169 (11.9)	
Insurance cost			< 0.0001
< 40,000 NTD	7618 (86.8)	31,052 (88.4)	
≥40,000 NTD	1159 (13.2)	4056 (11.6)	
Urbanization level			< 0.0001
Urban	6761 (77.0)	25,464 (72.5)	
Rural	2016 (23.0)	9644 (27.5)	
Living alone			0.62
No	7973 (90.8)	32,830 (90.7)	
Yes	804 (9.2)	3278 (9.3)	
Substance abuse			
No	8704 (99.2)	34,786 (99.1)	0.49
Yes	73 (0.8)	322 (0.9)	
Depression during the follow-up period	515 (5.9)	1282 (3.7)	< 0.0001

*SD* standard deviation, *NTD* New Taiwan Dollar

### Cumulative hazard curves by the Kaplan-Meier method

**Fig. 1:**
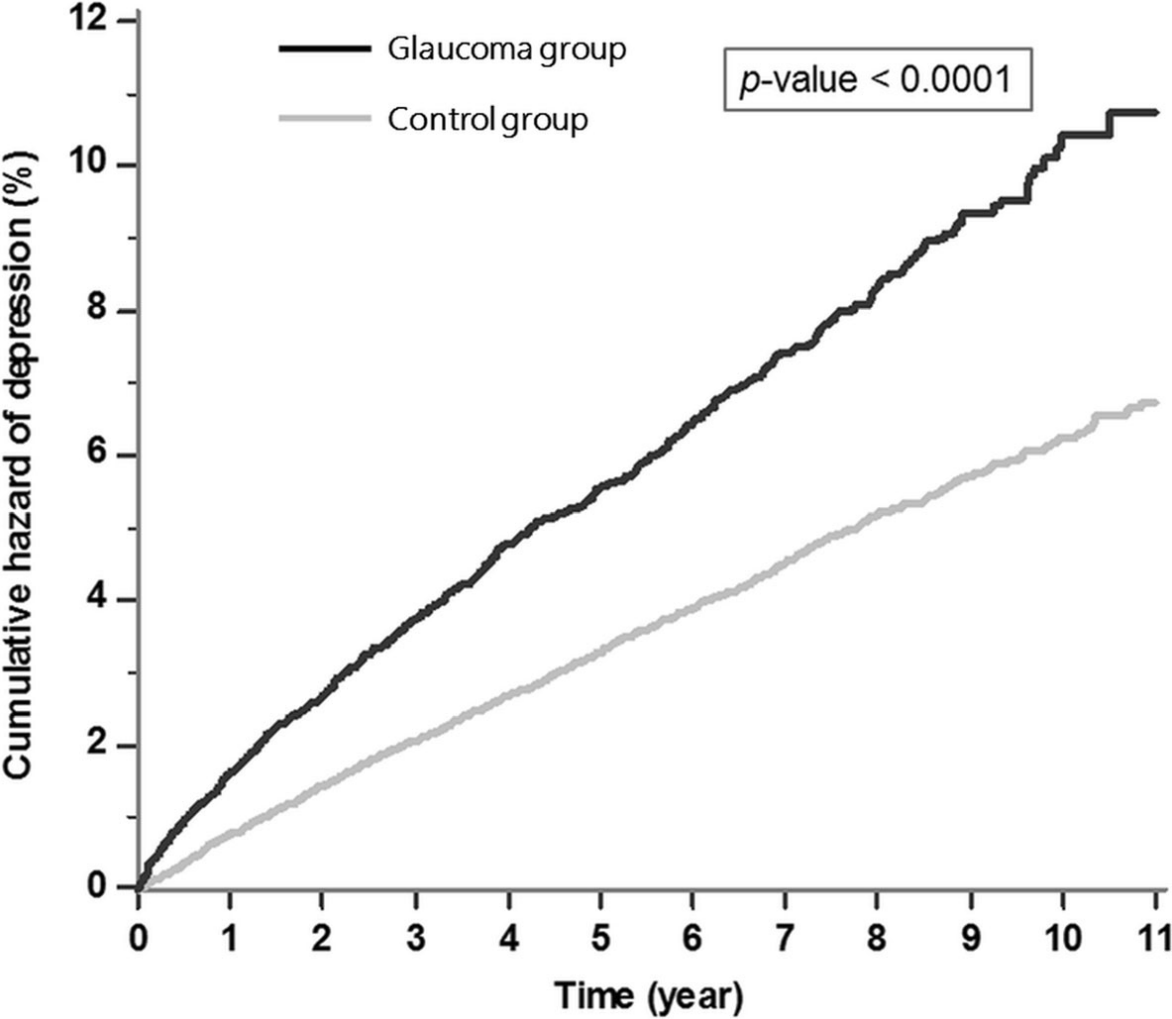
Kaplan-Meier curves for depression among glaucoma patients and the control group. The black line represents the glaucoma group and the gray line represents the control group

Figure 1 illustrates the cumulative hazard curves for depression in the glaucoma group and the control group. A log rank test revealed a statistically significant difference between the hazard curves of the two groups (*p*-value < 0.001).

### Univariate and multivariate analyses by the cox regression model

The unadjusted HR for depression was 1.71 times greater in the glaucoma group compared to the control group (95% confidence interval [CI]: 1.54–1.89) (Table 2). After adjusting for covariates, the significantly greater hazard for depression in the glaucoma group remained (adjusted HR = 1.71; 95% CI: 1.54–1.89). Age was a significant risk factor for depression in both univariate and multivariate analyses. The adjusted HR for depression in patients over 70 years old reached 1.49 when compared with those between 30 to 50 years old. Males were less likely to develop depression than females (adjusted HR = 0.67; 95% CI: 0.61–0.74). In the univariate analysis, patients with a greater CCI had a significantly higher risk of developing depression. However, after adjustment for covariates, CCI was no longer a significant risk factor (adjusted HR = 1.04; 95% CI: 0.91–1.19). Both living alone and substance abuse were significantly associated with an increased risk of depression (adjusted HR =1.20 and 1.44, respectively). The risk of developing depression was significantly lower in patients with a higher insurance cost (adjusted HR = 0.77; 95% CI: 0.64–0.92). However, residential urbanization, was not significantly related to the risk of depression in both univariate and multivariate analyses.

**Table 2:** Analyses of Risk Factors for depression in Patients with and without glaucoma

Predictive variables	Univariate analysis		Multivariable analysis	
	Unadjusted HR (95% CI)	*P* value	Adjusted HR (95% CI)	*P* value
Glaucoma (Yes vs. No)	1.71(1.54–1.89)	< 0.0001	1.71(1.54–1.89)	< 0.0001
Age				
30–50	Reference		Reference	
50–60	1.39(1.21–1.60)	< 0.0001	1.32(1.14–1.52)	< 0.001
60–70	1.53(1.34–1.75)	< 0.0001	1.40(1.21–1.61)	< 0.0001
≥70	1.61(1.41–1.85)	< 0.0001	1.49(1.29–1.72)	< 0.0001
Gender (Male vs Female)	0.64(0.59–0.71)	< 0.0001	0.67(0.61–0.74)	< 0.0001
Charlson comorbidity index				
< 3	Reference		Reference	
≥3	1.16(1.02–1.32)	< 0.05	1.04(0.91–1.19)	0.59
Insurance cost				
< 40,000 NTD	Reference		Reference	
≥40,000 NTD	0.61(0.51–0.73)	< 0.0001	0.77(0.64–0.92)	< 0.01
Urbanization level				
Urban	Reference		Reference	
Rural	1.03(0.93–1.14)	0.56	0.99(0.89–1.10)	0.85
Living alone				
No	Reference		Reference	
Yes	1.22(1.06–1.42)	< 0.01	1.20(1.03–1.41)	< 0.05
Substance abuse				
No	Reference		Reference	
Yes	1.54(1.09–2.18)	< 0.05	1.44(1.03–1.82)	< 0.05

*NTD* New Taiwan Dollar, *HR* hazard ratio, *CI* confidence interval,

In the multivariable analysis, all the other variables in the Table are included for adjustment

### Risk factors for depression among glaucoma patients

Table 3 displays the risk factors for depression among glaucoma patients. Older age, female gender, a lower insurance cost, living alone, and substance abuse, significantly increased the risk of developing depression among glaucoma patients in univariate as well as multivariate cox regression analyses. Neither residential urbanization nor the number of glaucoma medications were risk factors for depression. The use of β-blocker eye drops was not a significant risk factor for depression (adjusted HR = 1.08; 95% CI: 0.89–1.30). Similarly, none of other glaucoma medications was a risk factor or protective factor of depression.

**Table 3:** Analyses of Risk Factors for depression among Patients with glaucoma

Predictive variables	Univariate analysis		Multivariable analysis	
	Unadjusted HR	*p*-value	Adjusted HR	*p*-value
	(95% CI)		(95% CI)	
Age				
30–50	Reference		Reference	
50–60	1.56(1.23–1.99)	< 0.001	1.39(1.09–1.77)	< 0.001
60–70	1.31(1.03–1.68)	< 0.05	1.27(1.03–1.58)	< 0.05
≥ 70	1.56(1.22–1.99)	< 0.001	1.35(1.06–1.78)	< 0.05
Gender (Male vs. Female)	0.64(0.54–0.76)	< 0.0001	0.71(0.60–0.84)	< 0.0001
Charlson comorbidity index				
< 3	Reference		Reference	
≥ 3	1.18(0.94–1.49)	0.15	1.16(0.92–1.47)	0.23
Insurance cost				
< 40,000 NTD	Reference		Reference	
≥ 40,000 NTD	0.44(0.32–0.61)	< 0.0001	0.53(0.38–0.75)	< 0.001
Urbanization level				
Urban	Reference		Reference	
Rural	1.07(0.88–1.30)	0.49	0.97(0.80–1.18)	0.77
Living alone				
No	Reference		Reference	
Yes	1.36(1.06–1.73)	< 0.05	1.34(1.03–1.73)	< 0.05
Substance abuse				
No	Reference		Reference	
Yes	1.62(1.08–2.87)	< 0.05	1.51(1.02–2.57)	< 0.05
Number of glaucoma medications				
< 3	Reference		Reference	
≥ 3	0.88(0.70–1.11)	0.28	0.83(0.60–1.16)	0.28
Types of glaucoma medications				
Sympathomimetics (Yes vs. No)	0.91(0.76–1.08)	0.28	0.68(0.36–1.28)	0.23
Pilocarpine (Yes vs. No)	1.52(0.92–2.30)	0.11	1.57(0.93–2.10)	0.10
Carbonic anhydrase inhibitors (Yes vs. No)	0.82(0.67–1.01)	0.07	0.89(0.69–1.14)	0.35
β-blocker (Yes vs. No)	1.00(0.85–1.19)	0.97	1.07(0.88–1.29)	0.51
Prostaglandin analogs (Yes vs. No)	0.94(0.79–1.14)	0.54	1.40(0.74–2.68)	0.31

*NTD* indicates New Taiwan Dollar, *HR* indicates hazard ratio, *CI* indicates confidence interval, In the multivariable analysis, all the other variables in the Table are included for adjustment

## Discussion

We conducted an 11-year follow-up study on population-based data from the Taiwan LHID. Compared to those without glaucoma, patients with glaucoma had a significantly higher risk (HR = 1.71) of developing depression. Among glaucoma patients, older age, female gender, lower income, living alone, and substance abuse were significant risk factors for developing depression. Neither the number nor the types of glaucoma eye drops were significant risk factor of depression.

The majority of the prior studies regarding the association between glaucoma and depression used different kinds of self-report questionnaires to assess depression symptoms rather than a clinical diagnosis [4–10, 12, 13, 18], limiting comparisons between studies. In our study, diagnoses of glaucoma, depression, and systemic comorbidities were made by board-certificated doctors, increasing the validity of the results in the present study. Besides, the follow-up cohort design of our study could derive additional information about the incidence of depression. As noted in Table 1, during the 11-year follow-up period, the cumulative incidence of depression was 5.9% among glaucoma patients, which was significantly higher than the 3.2% in the control group.

According to the definition made by the World Health Organization, happiness/health is a dynamic state of complete physical, medical, social, and spiritual well-being. Therefore, systemic diseases, substance abuse, lack of social network such as family support or religion, all may have effects on depression risk. These variables are confounders and have to be adjusted when we investigate the relationship between glaucoma and depression. Table 1 compares these confounders between glaucoma and control groups. It is compatible with previous studies that the glaucoma group has a significantly higher severity of systemic diseases [5, 19]. Table 1 also shows the glaucoma group and the control group are matched well on age and gender, thus the two groups have the same distributions of age and gender. It is noteworthy that the mean glaucoma diagnostic age in our glaucoma group is 59.4 years, which is younger than the average glaucoma presenting age of 71.1 years in other country [20]. Besides, the diagnostic prevalence of glaucoma in the younger age categories was almost the same as that in the older age categories. One reason is that the prevalence of open-angle glaucoma (including normal tension glaucoma) has been higher than that of angle-closure glaucoma since 2005 [21], and patients with normal tension glaucoma generally have a younger age than those with angle-closure glaucoma [22], thus lowering the average age of glaucoma patients. Another reason is the high accessibility of healthcare achieved by the Taiwan NHI system [23], thus glaucoma can be diagnosed at an earlier age.

In addition, Table 1 shows that glaucoma subjects have a significantly higher insurance amount (income) as well as a significantly higher urbanization level of residence. The phenomenon can imply a higher chance of glaucoma to be identified in people with higher socioeconomic status (SES). The possible reason was that people with higher SES might have higher glaucoma awareness, and have more eye care seeking behavior because of increased prevalence myopia [24, 25]. Therefore they have more chances to be incidentally found to have glaucoma.

As shown in Table 2, the glaucoma group exhibited a significantly higher risk for developing depression than the control group (adjusted HR = 1.71). Similarly, a study conducted in the US by Wang et al., using the database of the National Health and Nutrition Examination Survey found glaucoma was significantly associated with depression after adjusting for the impact of demographic factors and systemic comorbidities (OR = 1.59; 95% CI: 1.01–1.52) [8]. Our cohort study design allowed an assessment of HR, providing more information than OR about subsequent risk of depression. We also found that lower income, living alone, and substance abuse were significant risk factors of depression, which were compatible with previous studies [26–29]. The possible explanation is the lack of financial or emotional support may predispose people to depression.

Among glaucoma patients in our study, older patients and female patients were at a greater risk of developing depression (Table 3). These results are consistent with previous studies [6, 10–12]. In addition, a lower income significantly increased the risk for depression, which implies that economic burden may predispose glaucoma patients to depression. Furthermore, living alone and substance abuse were associated with depression. Therefore, familial and social support were important for the psychological health among glaucoma patients. Our study revealed a consistent finding with previous studies that neither the number nor the types of glaucoma medications was a significant risk factor for depression among glaucoma patients [8, 30]. Also, we found the use of β-blocker eye drops was not an independent risk factors for depression. While systemic β-blockers are addressed to have side effects such as asthma, bradyarrhythmia, and depression [3, 31–33], the association between topical β-blocker eye drops and depression is still controversial. Topical β-blocker eye drops might be absorbed through nasal and pharyngeal mucosa and could cause depression in some studies [2, 34, 35] While drug manufactures label β-blockers with a possible risk of depression, the current study along with many prior studies [6, 8, 13, 36, 37] do not support this claim. The inconsistencies may be resulted from discrepancies in study design and case definition. Most of the studies supporting an association between β-blocker eye drops and depression used case series and case reports, whereas findings from cross-sectional or case-control studies were equivocal. Differences in case definition may also explain the inconsistencies. Studies using a diagnosis of depression mostly did not support the relationship. However, studies using depressive symptoms were about evenly split. Still another explanation of inconsistencies might be ethnicity. Most of previous literature revealed significant association were studies among Caucasians. Our study, with a cohort study design among an Asian population and used depression diagnosis as case definition, revealed no significant association between β-blocker eye drops and depression. Future studies will be needed to further investigate the issue using a comparable study design and case definition among different ethnicities.

The strengths of our study are a large sample size, a lengthy study period, disease confirmation through well-accepted ICD9-CM codes, and a statistical analysis that adjusted for confounders. However, studies based on claimed database may have the limitation that the diagnostic codes in the database are not as accurate as those in the clinical charts. Fortunately, in our health care system, the National health Administration (NHA) frequently checks the consistencies between the claimed data and the charts. NHA also checks whether the patient received a standard protocol of examinations to confirm the diagnoses. Therefore, the diagnoses in our database had high accuracies.

It is another limitation that NHIRD does not have the information regarding the detailed clinical characteristics of depression (e.g., Hamilton rating scale), or about visual quality. Besides, the NHIRD did not supply data about religion, which may have a protective effect on depression in previous studies [12, 38, 39]. Future studies combining the chart review will be conducted to consider and adjust these confounding effects. At present stage, we have adjusted many possible confounders, such as age, gender, CCI, income, urbanization level of residency, living alone, substance use, and number/types of glaucoma medications. Based on the significant risk factors of depression that were found in our study, early intervention to prevent or treat psychological problems could be considered among glaucoma patients with high risks.

## Conclusion

In our study, glaucoma patients are at significantly greater risk of developing depression. Among glaucoma patients, age, female, and low income were significant risk factors for depression. The findings from our study have clinical and public health implications. Clinically, when treating glaucoma patients, ophthalmologists need to focus not only on the medical aspects of glaucoma, but must also provide psychological support to their patients. Glaucoma patients at a high risk for depression, such as older patients, female patients, and patients with a low income, should be referred to a psychiatrist if early signs of depression become apparent. From a public health perspective, policy makers are encouraged to enforce screening for depression risk in patients with glaucoma, and to provide more substantial and integrated care.

## Abbreviations

ATC code: Anatomic Therapeutic Chemical code
CCI: Charlson comorbidity index (CCI)
CI: Confidence interval
HR: Hazard ratio
ICD-9-CM Codes: International Classification of Diseases, 9th Revision, Clinical Modification Codes
LHID: Longitudinal Health Insurance Database
NHA: National health Administration
NHI: National Health Insurance
NHIRD: National Health Insurance Research Database
SES: Socioeconomic status
